# Estimating Relatedness Between Malaria Parasites

**DOI:** 10.1534/genetics.119.302120

**Published:** 2019-06-17

**Authors:** Aimee R. Taylor, Pierre E. Jacob, Daniel E. Neafsey, Caroline O. Buckee

**Affiliations:** * Department of Epidemiology, Harvard T. H. Chan School of Public Health, Boston, Massachusetts 02115; † Broad Institute of MIT and Harvard, Cambridge, Massachusetts 02142; ‡ Department of Statistics, Harvard University, Cambridge, Massachusetts 02138; § Department of Immunology and Infectious Diseases, Harvard T. H. Chan School of Public Health, Boston, Massachusetts 02115

**Keywords:** identity-by-state, identity-by-descent, relatedness, independence model, hidden Markov model, malaria, *Plasmodium falciparum*, *Plasmodium vivax*, genetic epidemiology

## Abstract

Understanding the relatedness of individuals within or between populations is a common goal in biology. Increasingly, relatedness features in genetic epidemiology studies of pathogens. These studies are relatively new compared to those in humans and other organisms, but are important for designing interventions and understanding pathogen transmission. Only recently have researchers begun to routinely apply relatedness to apicomplexan eukaryotic malaria parasites, and to date have used a range of different approaches on an *ad hoc* basis. Therefore, it remains unclear how to compare different studies and which measures to use. Here, we systematically compare measures based on identity-by-state (IBS) and identity-by-descent (IBD) using a globally diverse data set of malaria parasites, *Plasmodium falciparum* and *P. vivax*, and provide marker requirements for estimates based on IBD. We formally show that the informativeness of polyallelic markers for relatedness inference is maximized when alleles are equifrequent. Estimates based on IBS are sensitive to allele frequencies, which vary across populations and by experimental design. For portability across studies, we thus recommend estimates based on IBD. To generate estimates with errors below an arbitrary threshold of 0.1, we recommend ∼100 polyallelic or 200 biallelic markers. Marker requirements are immediately applicable to haploid malaria parasites and other haploid eukaryotes. C.I.s facilitate comparison when different marker sets are used. This is the first attempt to provide rigorous analysis of the reliability of, and requirements for, relatedness inference in malaria genetic epidemiology. We hope it will provide a basis for statistically informed prospective study design and surveillance strategies.

GENETIC relatedness is a measure of recent shared ancestry (Weir *et al.* 2006; Speed and Balding 2015). It ranges from zero between two unrelated individuals to one between clones, and in the absence of inbreeding is broken down by recombination (Wright 1922). Since the early 20th century, relatedness has been used across a wide variety of fields: agriculture, forensic science, disease mapping, and ecology (Weir *et al.* 2006; Waples *et al.* 2019). Nevertheless, studies of relatedness are niche in the nascent field of infectious disease genetic epidemiology because only a subset of pathogens are eukaryotes, *e.g.*, helminths and parasitic protoza, which include malaria parasites (Gardy *et al.* 2015; Blanton 2018). Because relatedness is broken down by outbreeding, it can change with each generation (Thompson 2013). Studies of malaria parasite relatedness thus provide a sensitive measure of recent gene flow (Taylor *et al.* 2017), generating insight on an operationally relevant scale for disease control efforts (Blanton 2018; Wesolowski *et al.* 2018).

Malaria parasites are haploid during the human stages of their complex life cycle, which includes an obligate stage of sexual recombination between gametocytes within the mosquito (Baton and Ranford-Cartwright 2005). The probability of selfing depends on the number of parasite clones in the human source infection: certain if monoclonal *vs.* uncertain if polyclonal. Polyclonal infections result from either a single mosquito inoculation, in which case most parasite clones are likely interrelated, or multiple inoculations, in which case parasite clones are likely unrelated (Wong *et al.* 2017, 2018; Nkhoma *et al.* 2018). The prevalence of polyclonal infections depends on many epidemiological factors, *e.g.*, transmission intensity (Anderson *et al.* 2000; Schoepflin *et al.* 2009; Nkhoma *et al.* 2013) and correlates of human host immunity (Ntoumi *et al.* 1995; Konaté *et al.* 1999; Owusu-Agyei *et al.* 2002; Kiwuwa *et al.* 2013).

The diploid coefficient of inbreeding is a measure of relatedness between haploid genotype pairs, defined as a probability of identity-by-descent (IBD) (Hill 1996). Two alleles are identical-by-descent (also IBD) if descended from a recent common ancestor in some ancestral reference population (Bink *et al.* 2008; Thompson 2013; Speed and Balding 2015). IBD can also be interpreted in terms of shared segments unbroken by recombination since a recent common ancestor (Thompson 2013; Speed and Balding 2015), where the segment length distribution relates to ancestor generation under a coalescent model (Speed and Balding 2015). IBD segments underpin many applications from disease mapping (Browning and Thompson 2012) to *Plasmodium falciparum* selection detection (Henden *et al.* 2018), and can be averaged to generate a measure of relatedness (Speed and Balding 2015). However, coalescent interpretation remains challenging for malaria parasites because the complexities of their life cycle convolute generation in a setting-dependent manner. Two alleles that share the same allelic type are identical-by-state (IBS), and include those that are both IBD and not IBD but identical due to chance sharing of common alleles (Weir *et al.* 2006; Bink *et al.* 2008; Stevens *et al.* 2011; Thompson 2013; Huang *et al.* 2015; Speed and Balding 2015). While identity-by-state (also IBS) is observed, IBD is hidden and must be inferred.

Many estimators of relatedness exist, some assuming independence between IBD states (Weir *et al.* 2006; Bink *et al.* 2008) and others not [*e.g.*, Leutenegger *et al.* (2003) and subsequent models (Brown *et al.* 2012)]. Those assuming independence have fewer parameters but impaired power in the presence of dependence (Anderson and Garza 2006). Those that do not assume independence are often based on hidden Markov models (HMMs) (Rabiner 1989; Brown *et al.* 2012; Druet and Gautier 2017; Ramstetter *et al.* 2017). The HMM framework enables inference of IBD segments via one or more additional parameters that can be more difficult to reliably estimate than relatedness. Measures of relatedness used in studies of malaria include those estimated under HMMs [hmmIBD (Schaffner *et al.* 2018), isoRelate (Henden *et al.* 2018), and DEploidIBD (Zhu *et al.* 2018)]. IBS-based measures, *e.g.*, proportions of alleles shared [the haploid equivalent of the “allele-sharing coefficient” (Speed and Balding 2015)], or counts of allele differences, require only simple calculation and are thus popular also (Orjuela-Sánchez *et al.* 2009; Anderson *et al.* 2010; Daniels *et al.* 2015; Omedo *et al.* 2017a,b; Oyebola *et al.* 2018; Chang *et al.* 2019).

Despite many IBD- and IBS-based analyses, there are few systematic comparisons applicable to malaria studies. We compare IBD- and IBS-based measures for monoclonal malaria parasite samples using simulated data; various data sets of *P. falciparum*, the parasite responsible for the most-deadly type of human malaria; and a data set of *P. vivax*, the parasite most commonly responsible for malaria relapses. We use a framework encompassing two models assuming independence and not. It is an error-modified version of that of Leutenegger *et al.* (2003), which is at the core of many models (Brown *et al.* 2012), including those designed for comparison across malaria parasite samples (Henden *et al.* 2018; Schaffner *et al.* 2018). To guide future relatedness studies of malaria parasites and haploid eukaryotes more generally, we explore marker and allele counts for relatedness inference. We focus on relatedness alone, averaging over all IBD segments however small (Brown *et al.* 2012). Relatedness estimates are thus liable to reflect some linkage disequilibrium (LD) at the population level (Slatkin 2008). From relatedness alone, we can distinguish pairs that are highly related and not, but we cannot distinguish a highly inbred pair from an outbred pair with the same relatedness.

## Methods

### Relatedness

In this study, relatedness *r* is defined as the probability that, at any locus on the genome, the alleles sampled from two individuals are IBD. Let *m* denote the number of genotyped markers, each with a locus indexed by *t* = 1, . . . ,*m*. Let $c_{t}$ denote the index of the chromosome of the *t*-th locus, and $p_{t}$ its position on that chromosome (all markers are treated as point polymorphisms). For two indices $t_{1}<t_{2}$, we either have $c_{t_{1}}<c_{t_{2}}$, or $c_{t_{1}}=c_{t_{2}}$ and $p_{t_{1}}<p_{t_{2}}$. Let ${\text{IBD}}_{t}=1$ if two individuals are IBD at the *t*-th locus; otherwise ${\text{IBD}}_{t}=0$. We assume that *r* is constant across the genome: $r=P({\text{IBD}}_{t}=1)$ for all t=1,…,m. The sequence $\left({\text{IBD}}_{t}\right)$ could be made of independent variables, or could be a Markov chain, in which case, if we write $a_{j\ell }\left(t\right)$ for the probability of ${\text{IBD}}_{t}=\ell$ given that ${\text{IBD}}_{t-1}=j$, the model states

$$A\left(t\right)=\left(\begin{array}{cc} a_{00}\left(t\right) & a_{01}\left(t\right)\\ a_{10}\left(t\right) & a_{11}\left(t\right) \end{array}\right)$$

$$=\left(\begin{array}{cc} 1-r\left(1-\exp\left(-k\rho d_{t}\right)\right) & r\left(1-\exp\left(-k\rho d_{t}\right)\right)\\ \left(1-r\right)\left(1-\exp\left(-k\rho d_{t}\right)\right) & 1-\left(1-r\right)\left(1-\exp\left(-k\rho d_{t}\right)\right) \end{array}\right).$$

Above, $d_{t}$ denotes a genetic distance in base pairs between loci t−1 and *t*. If $c_{t-1}\neq c_{t}$, $d_{t}=\infty$; such that ${\text{IBD}}_{t-1}$ and ${\text{IBD}}_{t}$ are independent. The value k>0 parameterizes the switching rate of the Markov chain and ρ is a constant equal to the recombination rate, assumed fixed across the genome with value $7.4\times 10^{-7}\text{M}{\text{bp}}^{-1}$ for *P. falciparum* parasites (Miles *et al.* 2016).

The model connects *r* to the data as follows. At each locus, let $G_{t}=\left\{g_{1},\ldots ,g_{K_{t}}\right\}$ denote a set of alleles, where $K_{t}\geq 2$ denotes the cardinality of $G_{t}$ (allelic richness of the *t*-th marker). For individuals i,j at locus *t* we observe the pair $Y_{t}^{\left(i\right)},Y_{t}^{\left(j\right)}\in G_{t}$. We assume that alleles occur with frequencies ${\left(f_{t}\left(g\right)\right)}_{g\in G_{t}}$, with $f_{t}\left(g\right)>0$ for all $g\in G_{t}$ and $\sum_{l=1}^{K_{t}}f_{t}\left(g_{l}\right)=1$. The data comprise $Y_{t}^{\left(i\right)},Y_{t}^{\left(j\right)},d_{t}$, and ${\left(f_{t}\left(g\right)\right)}_{g\in G_{t}}$ at *m* loci. A simple observation model relates the data to ${\text{IBD}}_{t}$ by assuming that, if ${\text{IBD}}_{t}=0$, then $Y_{t}^{\left(i\right)}$ and $Y_{t}^{\left(j\right)}$ are independent categorical variables taking values in $G_{t}$ with probabilities ${\left(f_{t}\left(g\right)\right)}_{g\in G_{t}}$. If ${\text{IBD}}_{t}=1$, then $Y_{t}^{\left(i\right)}$ is such a categorical variable and $Y_{t}^{\left(j\right)}=Y_{t}^{\left(i\right)}$ with probability one. A more realistic model in Section B of File S1 accounts for observation error.

**Figure 1:**
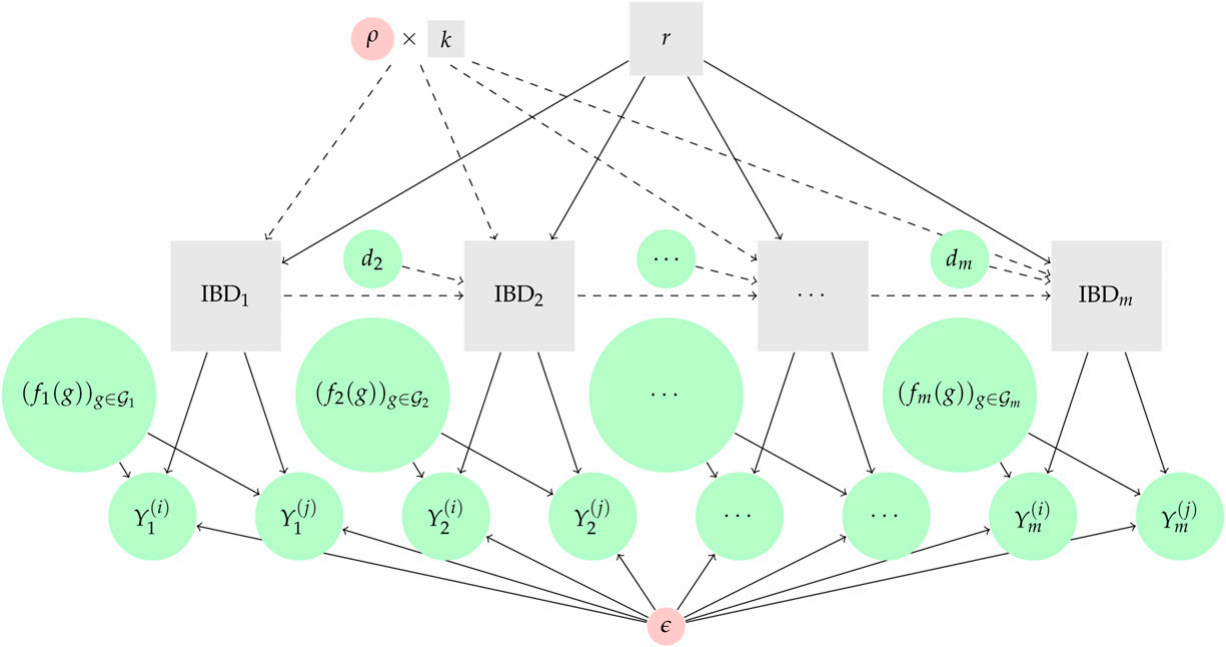
Models relating genetic data to genetic relatedness. Input data are depicted by green circles: for t=1…,m, genotype calls, $Y_{t}^{\left(i\right)}$ and $Y_{t}^{\left(j\right)}$, and allele frequencies, ${\left(f_{t}\left(g\right)\right)}_{g\in G_{t}}$; and for t=2…,m distances, $d_{t}$. Parameters considered fixed (genotyping error, *ϵ*, and constant, ρ) are depicted by red circles. Unobserved quantities are depicted by gray squares: IBD states, $\mathrm{IB}D_{1},\ldots ,\mathrm{IB}D_{m}$, and estimands *r* and *k*. Solid arrows depict dependencies under both the independence model and the HMM. Dashed arrows depict dependencies under the HMM only. HMM, hidden Markov model; IBD, identity-by-descent; IBS, identity-by-state.

Combining the Markov model for $\left({\text{IBD}}_{t}\right)$ with an observation model as above leads to an HMM (Figure 1) with likelihood function $\left(r,k\right)\mapsto {ℒ}_{1:m}\left(r,k\right)$, which can be evaluated using the forward algorithm. An independence model can be retrieved by setting $d_{t}=\infty$ for all *t*. Let ${\hat{r}}_{m}$ and ${\hat{k}}_{m}$ denote the maximum likelihood estimators (MLEs) of *r* and *k*, respectively. For each pair of individuals i,j, we compute them in R (R Core Team 2018). We use the one-dimensional optimize function to compute ${\hat{r}}_{m}$ under independence, and optim to compute ${\hat{r}}_{m}$ and ${\hat{k}}_{m}$ under the HMM, with initial values equal to 0.5 and 8, respectively. The default algorithm is that of Nelder and Mead (1965). Convergence of optim can be monitored via the number of calls made to the log-likelihood.

Under assumptions on the data-generating process, ${\hat{r}}_{m}$ could be shown to be consistent for *r* as m→∞. However, these asymptotic considerations are intricate in the present setting, where the degree of dependencies between observations increases with the sample size *m* due to decreasing intermarker distance (Hill and Weir 2011). This departs from standard asymptotic analysis where observations are not increasingly dependent as m→∞ (Douc and Moulines 2012); see also Section B of File S1.

Without standard results such as asymptotic normality of the MLE, there is no simple formula for sample size determination relating *m* to the variance of ${\hat{r}}_{m}$. The estimators’ distributions can still be approximately normal if the log-likelihood is approximately quadratic (Geyer 2013), in which case C.I.s can be obtained through the second derivative of the log-likelihood at the MLE. However, the present setting poses an additional difficulty since the MLE can be located on the boundary of the parameter space, ${\hat{r}}_{m}=0$ or ${\hat{r}}_{m}=1$ (Self and Liang 1987). Therefore, we rely on the parametric bootstrap (Wasserman 2013) to construct C.I.s around ${\hat{r}}_{m}$. Unless otherwise stated, we use 500 bootstrap draws throughout.

### Fraction IBS

For a pair of samples *i* and *j*, we define the fraction IBS as

$${\hat{\text{IBS}}}_{m}=\frac{1}{m}\sum_{t=1}^{m}{\text{IBS}}_{t}\text{where}{\text{IBS}}_{t}=1\mathrm{if}Y_{t}^{\left(i\right)}=Y_{t}^{\left(j\right)}\text{and zero otherwise}.$$ (1)

Its expectation is a linear function of relatedness, *e.g.*, when there is no genotyping error

$$E\left[{\hat{\text{IBS}}}_{m}\right]={\bar{h}}_{m}+(1-{\bar{h}}_{m})r,$$ (2)

where

$${\bar{h}}_{m}=\frac{1}{m}\sum_{t=1}^{m}h_{t}\text{and}h_{t}=\sum_{l=1}^{K_{t}}f_{t}{\left(g_{l}\right)}^{2}.$$ (3)

Here, $h_{t}$ and $1-h_{t}$ are equivalent to Nei’s gene identity and diversity, respectively, or, for an outbred diploid, homozygosity and heterozygosity, respectively, (Nei 1972, 1973; Nei and Tajima 1981). Equation 2 might suggest that ${\hat{\text{IBS}}}_{m}$ could converge to $\bar{h}+\left(1-\bar{h}\right)r$ (where $\bar{h}=\lim_{m\to \infty }{\bar{h}}_{m}$) as m→∞ under assumptions such as independent loci. Under this setup, the estimator ${\hat{\text{IBS}}}_{m}$ would not be consistent for *r*, but could be corrected (Section A of File S1).

### *Plasmodium* data

*P. falciparum* data are biallelic (*i.e.*, $K_{t}=2$ for all t=1,…,m) SNP data from monoclonal samples (Table 1). All data are published (Echeverry *et al.* 2013; Nkhoma *et al.* 2013; Cerqueira *et al.* 2017; Omedo *et al.* 2017a,b; Taylor *et al.* 2017). They were obtained either from sparse genome-wide panels of select markers, called barcodes, or from a dense whole-genome sequencing (WGS) data set; full details of sample collection and data generation can be found via the citations above, and references therein. Additional steps we took to process the data are as follows.

**Table 1:** A summary of globally diverse data sets of monoclonal *P. falciparum* samples

Data set and citation(s)*^a^*	Collection region and years	*n**^b^*	$m_{\text{max}}$*^c^*	${\bar{h}}_{m_{\text{max}}}$*^d^*	$\bar{K}\prime_{m_{\text{max}}}$*^e^*
Colombia (Echeverry *et al.* 2013)	Colombian Pacific region, 1993–2007	325	250	0.66	1.57
Thailand 93-SNP (Nkhoma *et al.* 2013; Taylor *et al.* 2017)	Thailand–Myanmar border, 2001–2010	1173	93	0.57	1.77
Thailand WGS (Cerqueira *et al.* 2017; Taylor *et al.* 2017)	Thailand–Myanmar border, 2001–2014	178	40210	0.89	1.16
The Gambia (Omedo *et al.* 2017a)	Kombo coastal districts, 2007–2008	71	31	0.77	1.37
Kilifi (Omedo *et al.* 2017a)	Coastal Kenya, 1998–2010	628	127	0.87	1.19
Western Kenya (Omedo *et al.* 2017b)	Western Kenya, 2008–2010	182	59	0.73	1.43

WGS, whole-genome sequencing.

a Full details of sample collection and data generation can be found via the citations above, and references therein. Additional steps we took to process the data for use in this study are described in section *Plasmodium* *data*.

b For each processed data set, *n* denotes the number of monoclonal *P. falciparum* samples.

c For each processed data set, $m_{\text{max}}$ denotes the maximum number of successfully genotyped SNPs per sample.

d For each processed data set, ${\bar{h}}_{m_{\text{max}}}$ denotes the expected homozygosity (Equation 3) averaged over $m_{\text{max}}$.

e For each processed data set, ${{\bar{K}}^{\prime }}_{m_{\text{max}}}$ denotes the effective cardinality (Equation 7) averaged over $m_{\text{max}}$.

Besides mapping SNP positions to the *P. falciparum* 3d7 v3 reference genome and recoding heteroallelic calls as missing [since all available samples were previously classified monoclonal (Echeverry *et al.* 2013)], we did not postprocess the Colombian data in any way. Thailand 93-SNP and WGS samples were used as described in Taylor *et al.* (2017). However, 5299 SNPs on chromosome 14 that were unintentionally omitted from the WGS data set in Taylor *et al.* (2017) are included here. Data derived from Omedo *et al.* (2017a,b) were processed using steps described in “Sample and SNP cut-off selection criteria” of Omedo *et al.* (2017a). In addition, we removed samples with duplicate SNP calls; removed samples classified as not monoclonal using a ≤5% heteroallelic SNP call rate to classify samples as monoclonal following (Nkhoma *et al.* 2013); and, among samples classified monoclonal, treated heteroallelic SNP calls as missing and removed monomorphic SNPs.

**Figure 2:**
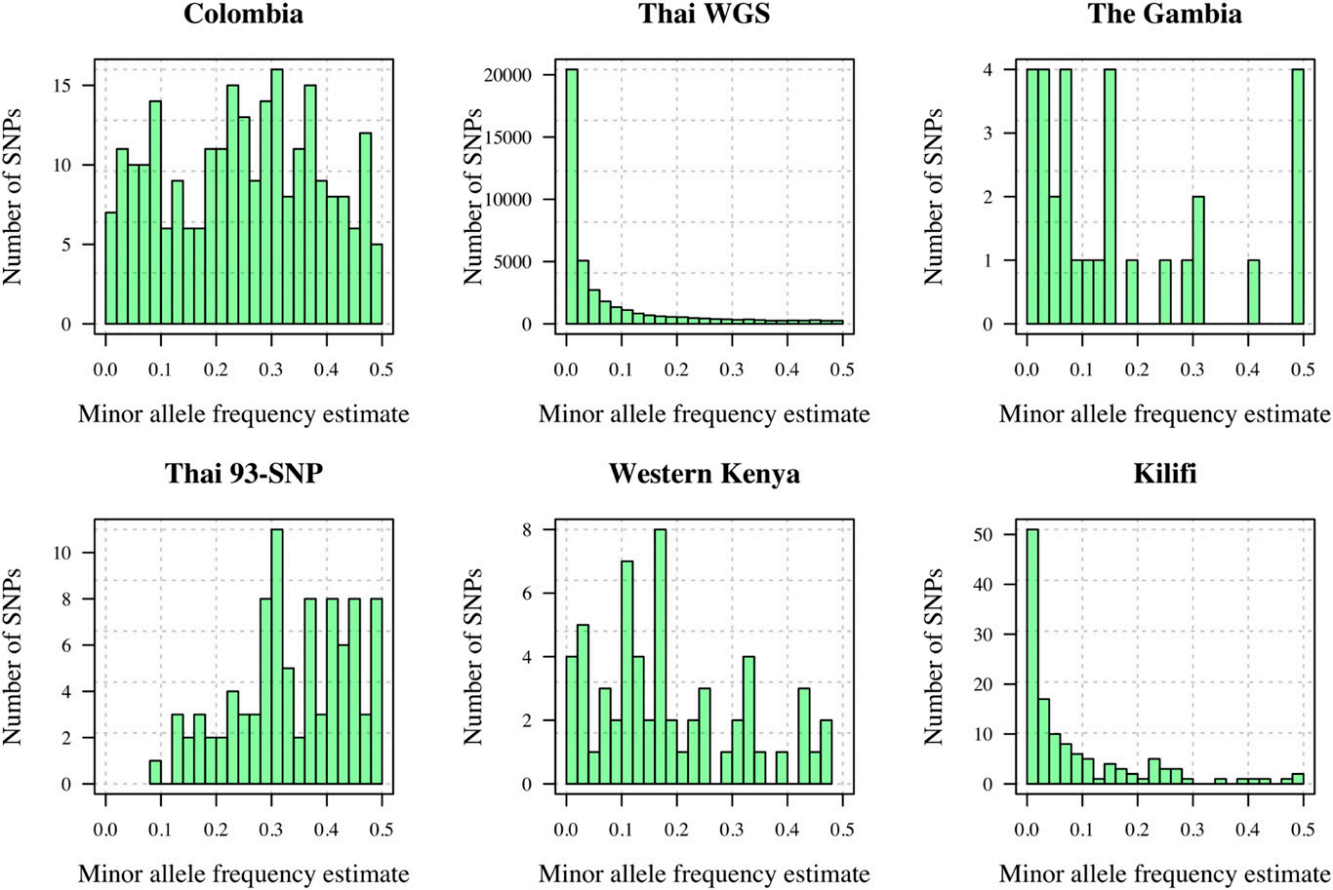
Minor allele frequency estimates from monoclonal *P. falciparum* data sets (Table 1). WGS, whole-genome sequencing.

For each *P. falciparum* processed data set, allele frequencies were estimated by simple proportions: $f_{t}\left(g_{l}\right)=n_{\text{nm}}^{-1}\sum_{i=1}^{n_{\text{nm}}}1\left(Y_{t}^{\left(i\right)}=g_{l}\right)$ for l=1,2 and each locus *t*, where $n_{\text{nm}}\leq n$ denotes the number of samples not missing data at the *t*-th locus. Minor allele frequencies, $\text{min}\left(f_{t}\left(g_{1}\right),f_{t}\left(g_{2}\right)\right)$, vary considerably due to different marker panels and spatiotemporal variation among parasite populations (Figure 2).

Samples in the *P. vivax* data set were collected between 2010 and 2014 from two clinical trials on the Thailand–Myanmar border (Chu *et al.* 2018a,b). They were genotyped at three to nine highly polyallelic microsatellites (MSs). In this study, we analyze samples genotyped at nine MSs with no evidence of polyclonality (detection of two or more alleles at one or more MS) from n=204 people, selecting one episode per person uniformly at random from all episodes per person. We use allele frequencies reported in Taylor *et al.* (2018). They have average expected homozygosity ${\bar{h}}_{m_{\text{max}}}=0.10$ and effective cardinality (defined below, Equation 7) averaged over $m_{\text{max}}=9$ MSs of ${{\bar{K}}^{\prime }}_{m_{\text{max}}}=13.03$. Since there are only nine markers, we analyze these data under the independence model.

### Simulated data

Unless otherwise stated, data were simulated under the HMM with genotyping error ε=0.001 using positions sampled uniformly from the Thailand WGS data set and with frequencies as follows. Biallelic marker data were simulated using frequencies sampled from the Thailand WGS data set with probability proportional to minor allele frequency estimates (to compensate for the skew toward rare alleles in WGS data set). Polyallelic marker data were simulated using frequencies sampled from a Dirichlet distribution using parameter vector α with $K_{t}$ entries each equal to 100 to generate frequencies for approximately equifrequent alleles, and α with entries each equal to 1 to generate frequencies uniform over the $K_{t}-1$ simplex, thus increasingly skewed toward rare alleles when $K_{t}>2$.

### Marker requirements for prospective relatedness inference

We explore marker requirements for error of ${\hat{r}}_{m}$ around *r*. By maximizing the likelihood we obtain estimates of both *r* and *k*, but we focus on the quality of the estimate of *r* only.

For a given setting [*e.g.*, *m*, *r*, *k*,$\left(K_{t}\right)$] we simulate 500 pairs of haploid genotype calls, and for each pair compute ${\hat{r}}_{m}$ and ${\hat{k}}_{m}$ under the HMM. We compute the root mean squared error (RMSE) of ${\hat{r}}_{m}$ around *r* over the 500 repeats. From the RMSEs, we derive *m* or $\left(K_{t}\right)$ required for RMSE under a prespecified value. Unless otherwise stated, when we fix *k* we use 8, the mean ${\hat{k}}_{m}$ for ${\hat{r}}_{m}\in \left(0.475,0.525\right)$ from the WGS data set; when we fix *r* we use 0.5, which we find leads to the largest RMSE, rendering data requirements based on r=0.5 conservative. For simplicity, we fix $K_{t}$ to be the same for all *t*. To explore *m* and $K_{t}$ for markers with and without equifrequent alleles, we use effective cardinality (Equation 7) averaged over all *m* considered,

$${{\bar{K}}^{\prime }}_{m_{\text{cum}}}=\frac{1}{m_{\text{cum}}}\sum_{t=1}^{m_{\text{cum}}}{K^{\prime }}_{t}\text{where}m_{\text{cum}}=24+96+192+288+384+480.$$ (4)

As an aside, comparison between ${\hat{r}}_{m}$ and *r* differs from that between ${\hat{r}}_{m}$ and “realized relatedness,” $L^{-1}\sum_{t=1}^{L}{\text{IBD}}_{t}$, where *L* is the length of the genome (Speed and Balding 2015). The former has the advantage of revealing RMSE due to the finite length of the genome [*i.e.*, Mendelian sampling (Hill and Weir 2011)], while at the same time revealing the excess and thus theoretically avoidable error due to marker limitations.

We consider the theoretical impact of $K_{t}>2$ at a single locus. For given $K_{t}$, we measure the informativeness via the Fisher information matrix (FIM), which relates to the precision of the MLE if the log-likelihood is approximately quadratic. We define ${\text{FIM}}_{t}=E\left[-\nabla_{r}^{2}\log P\left(Y_{t}^{\left(i\right)},Y_{t}^{\left(j\right)};r\right)\right]$, where the expectation is with respect to $Y_{t}^{\left(i\right)},Y_{t}^{\left(j\right)}$ given *r* and the allele frequencies; we assume no genotyping error for simplicity; the sign $\nabla_{r}^{2}$ stands for the second-order derivative with respect to *r*. ${\text{FIM}}_{t}$ depends on the allele frequencies ${\left(f\left(g_{l}\right)\right)}_{l=1}^{K_{t}}$ and on *r*:

$${\text{FIM}}_{t}\left(f_{t}\left(g_{1}\right),\ldots ,f_{t}\left(g_{K_{t}}\right),r\right)=\frac{1}{1-r}+\sum_{l=1}^{K_{t}}\left\{\frac{f_{t}\left(g_{l}\right){\left(1-f_{t}\left(g_{l}\right)\right)}^{2}}{r+f_{t}\left(g_{l}\right)\left(1-r\right)}-\frac{f_{t}{\left(g_{l}\right)}^{2}}{1-r}\right\}.$$ (5)

For any $K_{t}$ and *r*, it is maximized over all ${\left(f\left(g_{l}\right)\right)}_{l=1}^{K_{t}}$ by $f\left(g_{l}\right)=K_{t}^{-1}$ for all *l*, *i.e.*, by equifrequent alleles (proof in Section B of File S1), in agreement with high minor allele frequency (Thompson 1975). When alleles are equifrequent we obtain

$${\text{FIM}}_{t}\left(K_{t},r\right)=\frac{1}{1-r}+\frac{{\left(K_{t}-1\right)}^{2}}{K_{t}\left(1+\left(K_{t}-1\right)r\right)}-\frac{1}{K_{t}\left(1-r\right)}.$$ (6)

**Figure 3:**
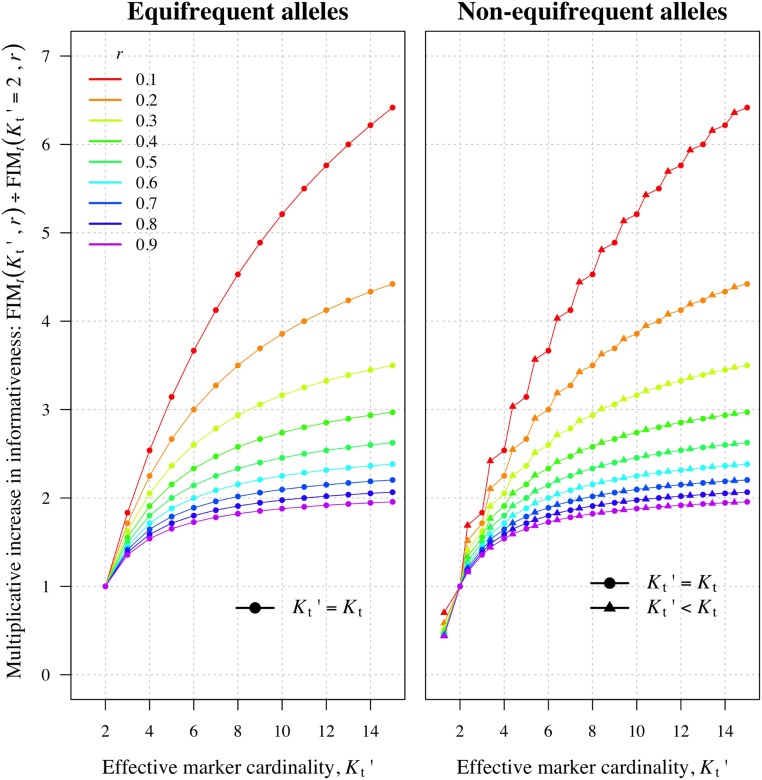
Multiplicative increase in the precision of the maximum likelihood estimator with marker cardinality. The left plot shows the multiplicative increase for equifrequent alleles according to Equation 6. The right plot shows the multiplicative increase with $K_{t}\prime$, where precision was calculated according to Equation 5 with either $f_{t}\left(g_{i}\right)=1/K_{t}\forall i=1,\ldots ,K_{t}$ (dots) or $f_{t}\left(g_{1}\right)=1.75/K_{t}$ and $f_{t}\left(g_{i}\right)=\left(1-f_{t}\left(g_{1}\right)\right)/\left(K_{t}-1\right)\forall i=2,\ldots ,K_{t}$ such that ${K^{\prime }}_{t}<K_{t}$ (triangles). FIM, Fisher information matrix.

To explore the theoretical gain of increasing $K_{t}>2$ we calculate the multiplicative increase in ${\text{FIM}}_{t}\left(K_{t}\geq 2,r\right)$ relative to ${\text{FIM}}_{t}\left(K_{t}=2,r\right)$ (Figure 3, left). The largest increase in precision is obtained upon increasing $K_{t}$ from 2 to 3 with increasing returns as *r* approaches zero. However the justification of the FIM as a measure of precision breaks at the boundary of the parameter space. The plot on the right of Figure 3 shows a multiplicative increase in precision as a function of effective cardinality,

$${K^{\prime }}_{t}=1/h_{t},$$ (7)

the noninteger number of equifrequent alleles concordant with $h_{t}$ based on the allele frequencies ${\left(f_{t}\left(g\right)\right)}_{g\in G_{t}}$. For example, ${K^{\prime }}_{t}=2$ is the effective cardinality of an “ideal” biallelic SNP with minor allele frequency 0.5, whereas ${K^{\prime }}_{t}<2$ is the effective cardinality of a realistic biallelic SNP with minor allele frequency <0.5. Precision increases with ${K^{\prime }}_{t}$ as it does with $K_{t}$.

### Data availability

All data used in this study are either simulated or published previously. Additional steps we took to process the data are described in section *Plasmodium data*. The processed data and code necessary for confirming the conclusions of the article are available at https://github.com/artaylor85/PlasmodiumRelatedness. All code was written in R (R Core Team 2018). Supplemental material available at FigShare: https://doi.org/10.25386/genetics.8977217.

## Results

This section is arranged as follows. First we consider the fraction IBS, ${\hat{\text{IBS}}}_{m}$, and show how it is problematic as an estimator of *r*. Second, we discuss ${\hat{r}}_{m}$ for *Plasmodium* data. Third, the performance of the HMM is compared to that of the independence model using simulated data. Fourth, we explore marker requirements for the estimation of *r* using simulated data.

### Fraction IBS as an estimator of relatedness

**Figure 4:**
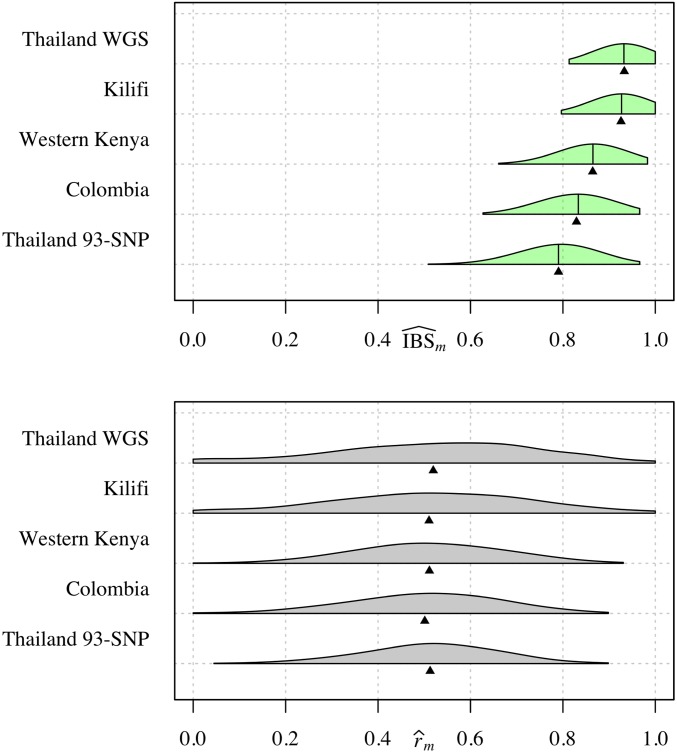
Measures of relatedness: parasite pairs simulated with relatedness 0.5. Half-violin plots showing distributions of ${\hat{\mathrm{IBS}}}_{m}$ (top) and ${\hat{r}}_{m}$ (bottom), each based on 1000 pairs simulated using r=0.5 and allele frequency estimates based on *P. falciparum* data sets with ≥ 59 SNPs (Table 1). To single out the effect of frequencies, we fixed all other parameters across the data sets including positions, which were extracted from the Western Kenyan data set. Allele frequencies were sampled uniformly at random from the full set of allele frequency estimates based on each data set. For each set of 59-SNP allele frequencies, the ${\bar{h}}_{m}$ values were 0.86, 0.85, 0.73, 0.67, and 0.58 (top to bottom row of each plot, respectively). Black vertical bars denote ${\bar{h}}_{m}+\left(1-{\bar{h}}_{m}\right)r$ (top), and triangles denote the mean ${\hat{\mathrm{IBS}}}_{m}$ (top) and mean ${\hat{r}}_{m}$ (bottom). IBS, identity-by-state; MS, microsatellite; WGS, whole-genome sequencing.

As an estimator of *r*, ${\hat{\text{IBS}}}_{m}$ does not satisfy favorable statistical properties but its expectation is a correlate of *r* (Equation 2). As such, studies have recovered trends in *r* (*e.g.*, with geographic distance) using IBS-based measures (Omedo *et al.* 2017a; Chang *et al.* 2019). However, quantitative trends and absolute values of ${\hat{\text{IBS}}}_{m}$ are only comparable across data whose markers have the same allele frequencies (Chang *et al.* 2019). To illustrate the effect of differing frequencies, we simulated ${\hat{\text{IBS}}}_{m}$ using r=0.5 and frequency estimates from published data sets (Figure 4, top). The ${\hat{\text{IBS}}}_{m}$ distributions are far from r=0.5 (we would expect to see bigger and smaller distances for data simulated using r<0.5 and r>0.5, respectively, with no difference for r=1). Their locations vary considerably, centering around ${\bar{h}}_{m}+\left(1-{\bar{h}}_{m}\right)r$ and rendering absolute values nonportable across data sets. In contrast, distributions of ${\hat{r}}_{m}$ all center around r=0.5 (Figure 4, bottom).

**Figure 5:**
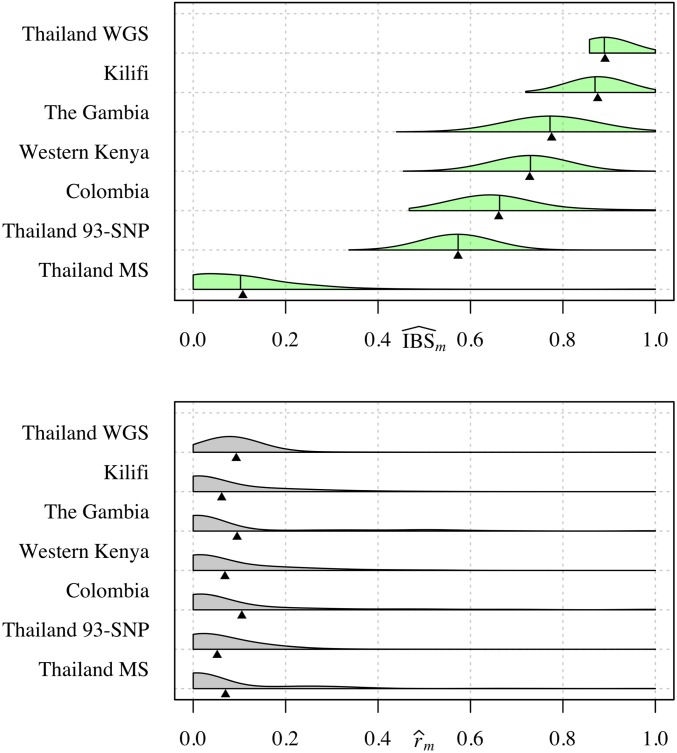
Measures of relatedness: parasite pairs with unknown relatedness. Half-violin plots showing distributions of ${\hat{\mathrm{IBS}}}_{m}$ (top) and ${\hat{r}}_{m}$ (bottom), based on pairwise comparisons of *Plasmodium* monoclonal samples from six published *P. falciparum* biallelic SNP data sets (Table 1) and a single *P. vivax* MS data set (Thailand MS). Black vertical bars denote ${\bar{h}}_{m_{\max}}$ (top), and triangles denote the mean ${\hat{\mathrm{IBS}}}_{m}$ (top) and mean ${\hat{r}}_{m}$ (bottom). IBS, identity-by-state; MS, microsatellite; WGS, whole-genome sequencing.

Figure 5 shows ${\hat{\text{IBS}}}_{m}$ and ${\hat{r}}_{m}$ distributions based on sample pairs from the published data sets. The locations and spreads of the ${\hat{\text{IBS}}}_{m}$ distributions vary considerably. They are not comparable across data sets, *e.g.,* among SNP data sets, the left-most centering of the Thailand 93-SNP distribution is not evidence that *P. falciparum* parasites from Thailand are less related than those from Kenya. Despite very different absolute values, each ${\hat{\text{IBS}}}_{m}$ distribution centers around ${\bar{h}}_{m_{\text{max}}}$, the ${\hat{\text{IBS}}}_{m}$ expectation when r=0. Thus, we conclude that many parasite pairs in these real data sets are unrelated, as corroborated by estimates based on IBD (Figure 5, bottom). The ${\hat{\text{IBS}}}_{m}$ distribution based on *P. vivax* data (Thailand MS) most closely approximates its partner ${\hat{r}}_{m}$ distribution due to highly polymorphic MSs.

### Relatedness of *Plasmodium* data

For each data set, ${\hat{r}}_{m}$ values range from 0 to 1, suggesting the presence of unrelated, partially related, and clonal parasites (Figure 5, bottom plot). However, the vast majority are <0.20. The skew toward lowly related parasite pairs is consistent with primary IBD-based analyses of the Thai *P. falciparum* data (Cerqueira *et al.* 2017; Taylor *et al.* 2017), as well as mean IBD fractions reported elsewhere (Zhu *et al.* 2018). Though the majority are <0.20, mean ${\hat{r}}_{m}$ values vary. Variation is caused by several factors. First, the mean is sensitive to small but variable counts of highly related parasite pairs: proportions of ${\hat{r}}_{m}>0.5$ range from 0.003 in the Thailand 93-SNP data set (lowest mean ${\hat{r}}_{m}$) to 0.062 and 0.065 in the Colombia and The Gambia data sets, respectively (highest mean ${\hat{r}}_{m}$). These highly related pairs are often the focus of demographic analyses, *e.g.*, (Chang *et al.* 2019). Considering largely unrelated pairs, some variation among data sets is likely due to LD. For example, among ${\hat{r}}_{m}<0.20$, the mean of the Thai WGS data set is 0.08, equal to the mean IBD fraction reported for Cambodia (0.08) and greater than that reported for Ghana (0.002) (Zhu *et al.* 2018). Overall, the interpretation and comparison of point estimates hinges on them being sufficiently precise; otherwise C.I.s facilitate comparison across different data sets.

**Figure 6:**
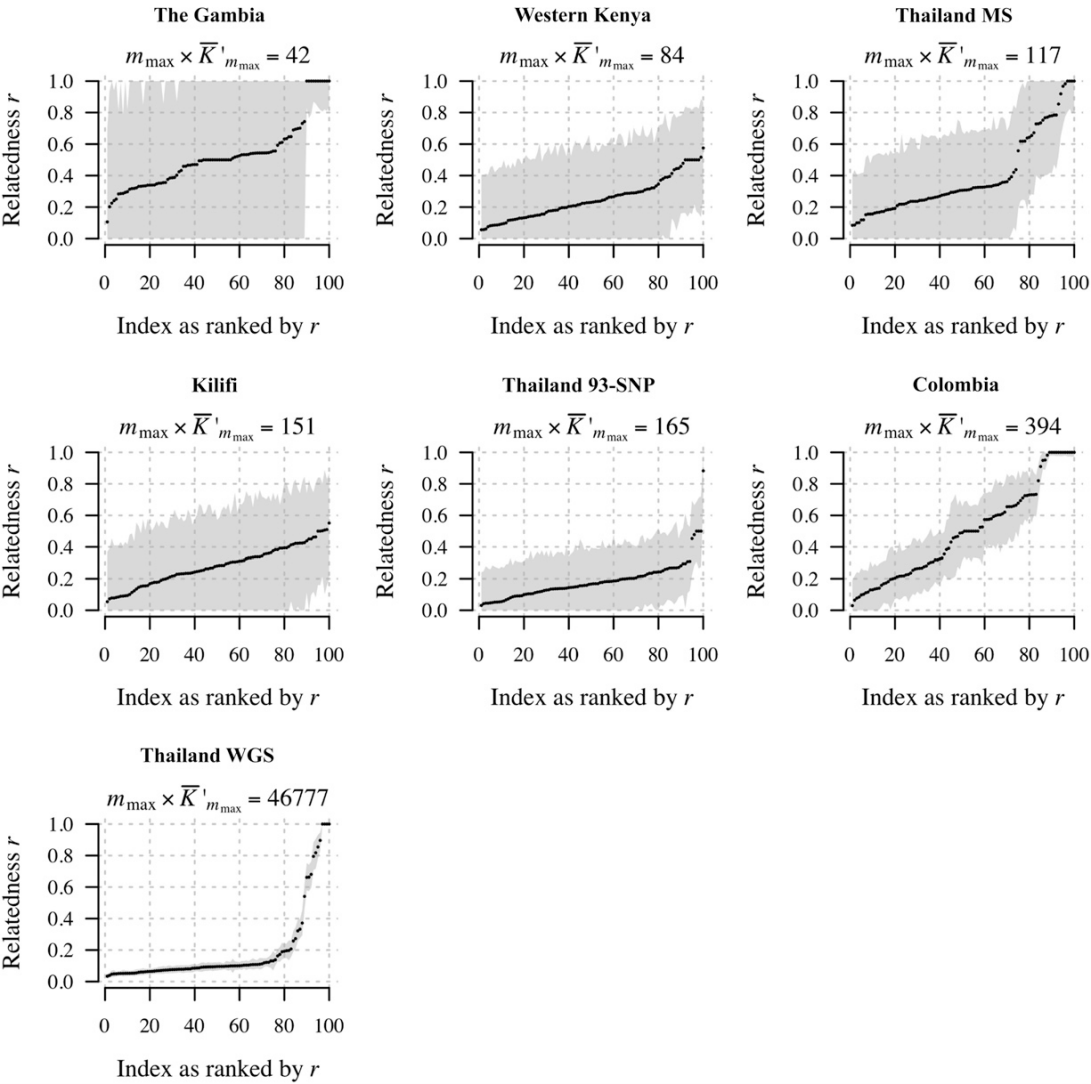
${\hat{r}}_{m}$ with 95% C.I.s for 100 select pairwise comparisons per data set of monoclonal *Plasmodium* samples from *P. falciparum* data sets (Table 1) and a single *P. vivax* data set, Thai MS.

For 100 estimates selected specifically to span the [0, 1] range, Figure 6 shows 95% C.I.s. In general, they are tighter around estimates for data sets with larger $m_{\text{max}}\times {{\bar{K}}^{\prime }}_{m_{\text{max}}}$, an observation we will return to. Considering the boundaries, intervals around estimates of *r* close to 1 are tighter, in general, than those for *r* close to 0. Due to the nonquadratic nature of the log-likelihood of *r* when ${\hat{r}}_{m}$ is close to either 0 or 1 (*e.g.*, Figure B.3 of File S1, left top and middle), we construct C.I.s using the parametric bootstrap. For ${\hat{r}}_{m}$ away from 0 and 1, the log-likelihood is quadratic (*e.g.*, Figure B.3 of File S1, bottom left plot) and thus normal approximation C.I.s could be constructed. As an aside, Figure B.3 also demonstrates both the difficulty in estimating *k* and the robustness of ${\hat{r}}_{m}$ relative to ${\hat{k}}_{m}$ when ${\hat{r}}_{m}$ is close to the boundaries.

### The HMM *vs.* the independence model

**Figure 7:**
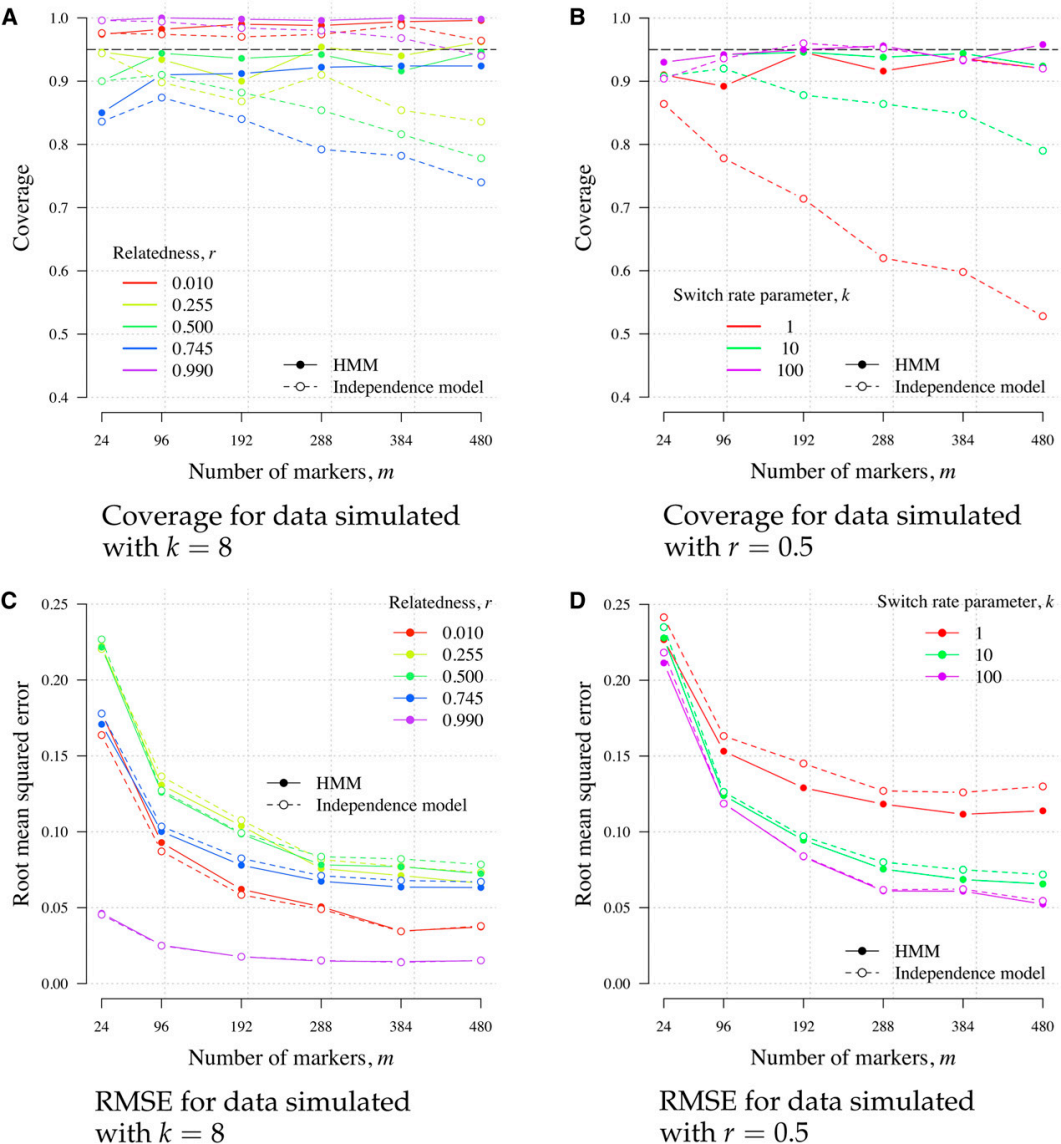
Coverage (panels A and B) and RMSE (panels C and D) under the HMM and the independence model. Coverage is equal to the proportion of 500 ${\hat{r}}_{m}$ whose 95% parametric bootstrap C.I.s contain the value of *r* used to simulate the data. Data were simulated under the HMM with ε=0.001, $K_{t}=2$ for all *t*, *k* = 8 for various *r* (panels A and C), and *r* = 0.5 for various *k* (panels B and D). HMM, hidden Markov model; MS, microsatellite; RMSE, root mean squared error.

The HMM was used to compute ${\hat{r}}_{m}$ for biallelic *P. falciparum* data sets, all of which have $m_{\text{max}}>24$ (Table 1), whereas the independence model was used for the polyallelic *P. vivax* data set, Thai MS, whose $m_{\text{max}}=9$. In this section, the performance of the HMM is compared to that of the independence model using data simulated under the HMM. The main difference between the HMM and the independence model is estimation uncertainty. Under a well-specified model, 95% C.I.s should have 95% coverage, *i.e.*, contain the value of *r* used to simulate the data 95% of the time. The HMM provides coverage close to 0.95 for m>24, while the independence model (misspecified) provides waning coverage for m>24, especially when *k* is small. For m=24, both the HMM and the independence model provide similar coverage, above or around 0.85 (Figure 7, a and b). In terms of *r* estimation accuracy, the two models are similar, with only a slight increase in RMSE under the independence model when k≤10 (Figure 7, c and d). The computational cost of obtaining the MLE under either model is comparable; timings are provided in Section B of File S1.

### Marker requirements for prospective relatedness inference

As Figure 4 exemplified using simulated data, estimates of ${\hat{r}}_{m}$ concentrate around the value of *r* used to simulate the data. However, in Figure 4 they do so with large variability, due to limited data (m=59 with $K_{t}=2\forall t$). We now consider how large *m* needs to be to estimate *r* with specified RMSE, first considering biallelic markers with $K_{t}=2$ for all *t*, and second considering polyallelic markers with $K_{t}\geq 2$.

#### Biallelic markers:

**Figure 8:**
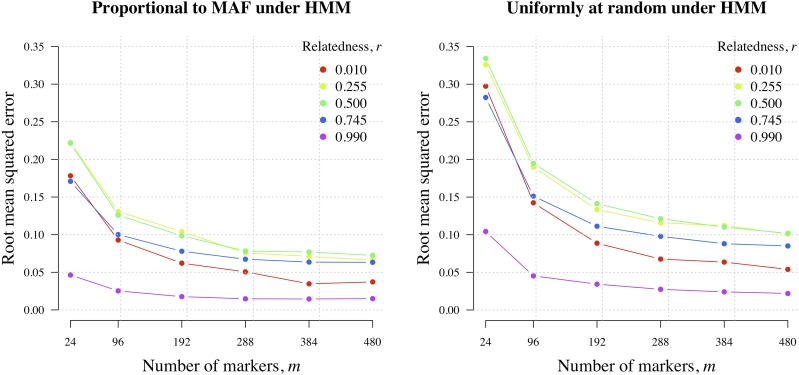
RMSE of ${\hat{r}}_{m}$ generated under the HMM. Data were simulated under the HMM using various *r* (see legend); allele frequencies drawn from the WGS data set with probability proportional to their MAFs (${\bar{h}}_{m}\approx 0.69$ and ${{\bar{K}}^{\prime }}_{m}\approx 1.53$, left plot) and uniformly at random (${\bar{h}}_{m}\approx 0.89$ and ${{\bar{K}}^{\prime }}_{m}\approx 1.17$, right plot) (values are approximate due to some variation across *m*). HMM, hidden Markov model; MAF, minor allele frequency; RMSE, root mean squared error.

Biallelic markers include biallelic SNPs, the most abundant polymorphic marker type, commonly used for relatedness inference (Weir *et al.* 2006). Figure 8 shows the RMSE of **${\hat{r}}_{m}$** generated under the HMM given allele frequencies drawn from the WGS data set, with probability proportional to their minor allele frequencies *vs.* allele frequencies drawn uniformly at random. Errors obtained using the former approach are smaller (Figure 8, left) in agreement with the long-established result that higher minor allele frequencies are preferable for relationship inference (Thompson 1975). Either way, RMSE is relatively large for 24 markers, decreasing dramatically when **m=96**, with diminishing returns thereafter (it does not tend to zero due to the finite length of the genome). Also of note, RMSE decreases with increasing proximity of the data-generating *r* to either 0 or 1 (especially the latter). As such, biallelic marker requirements for inference of **r=0.5** constrain guidelines for inference of *r* in general (Table 2).

**Table 2:** Biallelic marker requirements for specified RMSE around specified *r*

RMSE	r=0.01	r=0.50	r=0.99	Any r∈(0,1)*^a^*
0.00	>L*^b^*	>L	>L	>L
0.05	480–288	>480	< 24	> 480
0.10	24–96	96–192	< 24	192
0.15	24–96	24–96	< 24	96
0.20	< 24	24–96	< 24	96

Data extracted from Figure 8, left. RMSE, root mean squared error.

a Since r=0.5 has the largest marker requirements in general, inference of any r∈(0,1) is given by the maximum of the marker requirement interval for r=0.5.

b The length of the genome is denoted by *L*.

#### Polyallelic markers:

Highly polyallelic MS markers have long been used for relatedness inference and there is growing interest in using microhaplotypes (regions of high SNP diversity, unbroken by recombination) (Weir *et al.* 2006; Baetscher *et al.* 2018). Neither MSs nor microhaplotypes are point polymorphisms. However, to explore the general utility of polyallelic markers for relatedness inference, we make the simplifying assumption that they are. We focus on r=0.5, since for biallelic markers r=0.5 had the largest marker requirements in general (Table 2).

**Figure 9:**
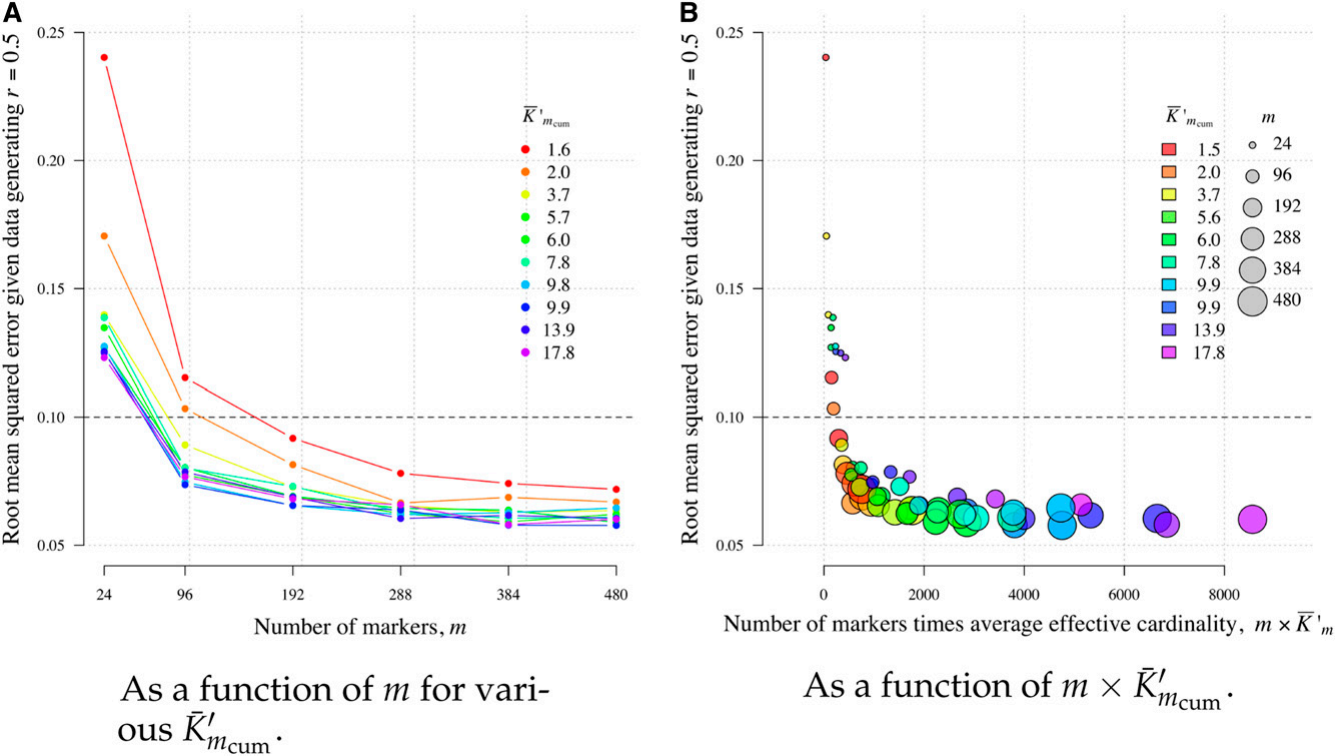
Root mean squared error of ${\hat{r}}_{m}$ around data generating r=0.5 as a function of *m* for various $\bar{K}\prime_{m_{\text{cum}}}$ (panel A) and as function *m* × $\bar{K}\prime_{m_{\text{cum}}}$ (panel B).

Figure 9a shows three notable results. First, if only a small number of markers (*e.g.*, 24) are available, a slight increase in their average effective cardinality markedly reduces RMSE, with diminishing returns as *m* grows. Second, to obtain RMSE less than some arbitrary amounts, one can either increase cardinality or *m*. For example, to obtain RMSE <0.1, our results suggest typing 96 markers with ${{\bar{K}}^{\prime }}_{m}>2$ or ∼192 markers with ${{\bar{K}}^{\prime }}_{m}=1.6$ (concordant with Table 2). However, third, within the range of *m* values explored here, markers with $K_{t}>2$ are necessary for optimally low RMSE (*i.e.*, RMSE comparable with Mendelian sampling).

The results shown in Figure 9a are projected onto a single axis in Figure 9b. Larger $m\times \bar{K}\prime_{m_{\text{cum}}}$ provides smaller RMSE with diminishing returns beyond $m\times \bar{K}\prime_{m_{\text{cum}}}\approx 2000$. Informally, this result provides intuition as to why we obtain, in general, tighter C.I.s around ${\hat{r}}_{m}$ based on *Plasmodium* data sets with larger $m_{\text{max}}\times \bar{K}\prime_{m_{\text{max}}}$ (Figure 6). Moreover, it suggests that the C.I.s around the Thailand WGS estimates are as small as they can be.

## Discussion

Using a simple model framework, we call attention to properties of estimates of genetic relatedness, *r*, increasingly used in genetic epidemiology of malaria. These results are applicable more generally to haploid eukaryotes, while highly recombining prokaryotes would require model modifications.

The fraction IBS is a simple data statistic that includes the chance sharing of common alleles (Thompson 2013). It is not a statistically principled estimator of *r*. As such, it does not allow calculation of C.I.s for *r*, nor marker requirements. Its expectation is a correlate of *r*, but absolute values and quantitative trend estimates are not portable across studies due to dependence on allele frequencies, which vary in space and time, and with different marker panels and quality control procedures (Speed and Balding 2015). However, it is simple and its use will persist. To aid interpretation across studies that continue using IBS-based measures to investigate relatedness, we show how it is expected to change as a function of *r* and allele frequencies.

Model-based relatedness inference allows construction of C.I.s and marker requirements. Based on the parameters we explored, to achieve error arbitrarily below 0.10, data for ≥ 200 biallelic or 100 polyallelic markers are recommended (fewer are required if markers are highly polyallelic). In practice, a set of makers could combine different marker types.

We present results based on a gobal set of published data sets. The original studies all feature relatedness estimates either based on allele sharing (Echeverry *et al.* 2013; Nkhoma *et al.* 2013), SNP differences (Omedo *et al.* 2017a,b), or IBD (Cerqueira *et al.* 2017; Taylor *et al.* 2017). Those using IBS-based estimates legitimately focus on a single data set, recovering meaningful but data set-specific quantitative results. Where comparisons can be made, our results generally agree with primary analyses (Table C.1 of File S1). More widely, our results agree (in order of magnitude) with those reported for diploids and polyploids (Table C.2 of File S1). Relatedness inference for polyploids [*e.g.*, (Wang and Scribner 2014; Huang *et al.* 2015)] is similar to that for polyclonal malaria samples. However, the latter is more challenging, since the equivalence of ploidy is unknown and variable. Despite these challenges, methods to infer relatedness within polyclonal malaria samples exist (Henden *et al.* 2018; Zhu *et al.* 2018), while methods to infer relatedness across polyclonal malaria samples are under development. It will be interesting to see how marker requirements for monoclonal samples scale in this more complex setting.

Our results are limited by various simplifying assumptions; most problematically, fixed allele frequencies (Speed and Balding 2015; Waples *et al.* 2019). Typically, allele frequencies are estimated using data intended for relatedness inference yet assuming independent and identically distributed samples (Wang 2004; Voight and Pritchard 2005). These data-derived allele frequencies can lead to relatedness underestimation (Bink *et al.* 2008). Improving them could benefit inference more than increasing the number of markers (Bink *et al.* 2008). To better estimate malaria parasite allele frequencies, one could jointly model frequencies and relatedness (Wang 2004). Moreover, by borrowing information across samples and extending the inference framework, one could theoretically infer the ancestral recombination graph and thus the malaria parasite genetic map (presently assumed uniform across the malaria parasite genome (Henden *et al.* 2018; Schaffner *et al.* 2018; Zhu *et al.* 2018)). That said, complexities specific to malaria (*e.g.*, selfing and its association with transmission) present unique challenges (Speidel *et al.* 2019). Modular multi-way extensions of pairwise methods may also outperform pairwise methods (Ramstetter *et al.* 2018).

Formally stated in Equation 6, a highly polyallelic marker can be several times more informative than a biallelic marker for relatedness inference, as for population assignment (Rosenberg *et al.* 2003). Despite superior informativeness, MSs are being superceded by SNPs for relatedness inference, due to the abundance, and relative ease and reliability of typing SNPs (Weir *et al.* 2006). Microhaplotypes combine the ease of SNPs with the informativeness of polyallelic markers (Baetscher *et al.* 2018). They can be defined using an LD-based decision theoretic criterion (Rosenberg *et al.* 2003; Slatkin 2008; Gattepaille and Jakobsson 2012), and genotyped using amplicon sequencing (Neafsey *et al.* 2015; Baetscher *et al.* 2018) or molecular inversion probes (MIPs), also used to genotype MSs and SNPs (Mu *et al.* 2010; Hiatt *et al.* 2013; Aydemir *et al.* 2018). Amplicon and MIP approaches are especially useful given polyclonal samples, because they can capture within-host clonal densities and phases (Neafsey *et al.* 2015; Aydemir *et al.* 2018). A model that accurately reflects the fact that MSs and microhaplotypes are not point polymorphisms, while accounting for their associated mutation and observation error rates, merits consideration (Hoffman and Amos 2005; McDew-White *et al.* 2019).

Besides motif repeats within MSs and SNPs within microhaplotypes (presently overlooked), it is preferable to minimize dependence between markers. Dependence is a function of marker position and LD. When considering polyallelic markers, we sampled marker positions uniformly at random from the Thailand WGS data set. For microhaplotypes, a more realistic approach would draw from genomic intervals amenable to physical phasing and with high within-interval LD. If diverse windows are genomically clustered, this presents a trade-off between distance and cardinality. We do not consider the trade-off here, but it can be explored within the current framework and is a topic of future work. Regarding LD, some models commonly used in human genetics account for it (Browning 2008; Browning and Browning 2010) [also see Brown *et al.* (2012)], but those designed to estimate relatedness between malaria parasites do not (Henden *et al.* 2018; Schaffner *et al.* 2018; Zhu *et al.* 2018). LD reported in malaria parasite populations is highly setting-dependent but generally lower than that reported in human populations (Anderson *et al.* 2000; International HapMap Consortium *et al.* 2007; Neafsey *et al.* 2008; Echeverry *et al.* 2013; Samad *et al.* 2015). Its incorporation into methods for malaria parasite relatedness inference, both within and between polyallelic markers, warrants further research.

Here and elsewhere (Table C.2. of File S1), marker requirements are based on either down-sampled or simulated data. Standard asymptotic theory for HMMs is problematic in the present setting due to the finite length of the genome, and the increasing degree of dependencies between markers as their density grows. Understanding the finite sample properties of the MLE in this setting remains an open problem. Another open problem beyond the scope of this study, is that of sampling individuals for population-level inference (*e.g.*, how many parasite samples are required to reliably infer gene flow between different geographic locations using relatedness?). Work is ongoing to address these questions, which are very application-specific and dependent on many population factors (*e.g.*, transmission intensity, seasonality, and asymptomatic reservoir).

### Conclusion

For portability, we recommend estimates of relatedness based on IBD for malaria epidemiology. To generate estimates between monoclonal parasite samples with r=0.5 (which we find leads to the largest error) with < 0.1 error, ∼200 biallelic or 100 polyallelic markers are required. C.I.s facilitate comparison across studies that inevitably differ in terms of available genetic data. Together with anticipated work on population-level sampling, we hope this work on genetic-level sampling (and extensions thereof) will aid statistically informed design of prospective genetic epidemiological studies of malaria.
